# Proteomic Characterization of Plasma Rich in Growth Factors and Undiluted Autologous Serum

**DOI:** 10.3390/ijms222212176

**Published:** 2021-11-10

**Authors:** Eduardo Anitua, Francisco Muruzabal, Ander Pino, Roberto Prado, Mikel Azkargorta, Felix Elortza, Jesús Merayo-Lloves

**Affiliations:** 1 BTI—Biotechnology Institute, 01007 Vitoria, Spain; francisco.muruzabal@bti-implant.es (F.M.); ander.pino@bti-implant.es (A.P.); roberto.prado@bti-implant.es (R.P.); 2 University Institute for Regenerative Medicine and Oral Implantology—UIRMI (UPV/EHU-Fundación Eduardo Anitua), 01007 Vitoria, Spain; 3 Proteomics Platform, CIC bioGUNE, CIBERehd, ProteoRed-ISCIII, Bizkaia Science and Technology Park, 48160 Derio, Spain; mazkargorta@cicbiogune.es (M.A.); felortza@cicbiogune.es (F.E.); 4 Instituto Oftalmológico Fernández-Vega, Fundación de Investigación Oftalmológica, Universidad de Oviedo, 33012 Oviedo, Spain; merayo@fio.as

**Keywords:** platelet-rich plasma, PRP, plasma rich in growth factors, PRGF, autologous serum, AS, proteomic, keratocytes, cornea, ocular surface

## Abstract

Over the last three decades, there has been special interest in developing drugs that mimic the characteristics of natural tears for use it in the treatment of several ocular surface disorders. Interestingly, the composition of blood plasma is very similar to tears. Therefore, different blood-derived products like autologous serum (AS) and plasma rich in growth factors (PRGF) have been developed for the treatment of diverse ocular pathologies. However, scarce studies have been carried out to analyze the differences between both types of blood-derived products. In the present study, blood from three healthy donors was drawn and processed to obtain AS and PRGF eye drops. Then, human corneal stromal keratocytes (HK) were treated with PRGF or undiluted AS. Proteomic analysis was carried out to analyze and characterize the differential protein profiles between PRGF and AS, and the differentially expressed proteins in HK cells after PRGF and AS treatment. The results obtained in the present study show that undiluted AS induces the activation of different pathways related to an inflammatory, angiogenic, oxidative stress and scarring response in HK cells regarding PRGF. These results suggest that PRGF could be a better alternative than AS for the treatment of ocular surface disorders.

## 1. Introduction

The increase in life expectancy across the last few decades is associated with the higher incidence of age-related diseases, including those pathologies affecting all tissues composing the eye from the ocular surface to the eye fundus.

Artificial tears are commonly used for the topical treatment of several ocular surface disorders. However, the physico-chemical properties of natural tears are by far more complex than artificial tears including pH, osmolarity and their complex composition of water, lipids, proteins or salts among other components [1]. Several other treatments, like anti-inflammatory substances (corticoids or cyclosporine or secretagogues), have shown an improvement in symptoms, but it has been demonstrated that their use could induce side effects, such as ocular burning or increased ocular pressure [2,3,4]. New and interesting regenerative therapies like amniotic membrane transplantation (AMT) and recombinant growth factors have been used for the treatment of several ocular surface pathologies in the ophthalmology field [5]. However, the use of these treatments presents some limitations, such as the uncertain results, the elevated manufacturing costs, and the risk of disease transmission in the case of AMT, making it necessary to explore other therapeutic approaches for ocular surface tissue regeneration [6,7,8].

Over the last thirty years, there has been a special interest in developing drugs that mimic the characteristics of natural tears such as the regenerative, lubrication, antimicrobial and anti-inflammatory properties. Interestingly, the composition of blood plasma is very similar to tears in terms of osmolarity, pH and many proteins related to tissue regeneration [9,10,11]. In this sense, different blood-derived products like autologous serum (AS) and platelet-rich plasma (PRP) have been developed for the treatment of diverse ocular pathologies like dry eye disease, persistent epithelial defects, or corneal ulcers with satisfactory results [12]. Nonetheless, the manufacturing protocol and, as a consequence, the composition of AS products, has been changing over time, mainly in relation to their preparation, such as centrifugation, coagulation time or dilution. This lack of protocol standardization, together with its high content in pro-inflammatory molecules like metalloproteinases and hydrolases, has provided controversial AS clinical outcomes [11,13]. In order to avoid these limitations, a protocolized and standardized technology has been developed: plasma rich in growth factor (PRGF) eye drops. This is a PRP with specific features that include platelet activation, avoiding leukocytes and pro-inflammatory molecules, and which contain a higher content of growth factors than AS [14,15,16]. These differences have been widely observed in several clinical studies where PRGF advantages have been shown in corneal epithelial defects, dry eye, neurotrophic keratitis and graft versus host diseases that were refractory to previous treatment with AS [17,18,19].

Furthermore, several pre-clinical studies have demonstrated the different capabilities of AS and PRGF to induce differential biological activities in ocular surface cells. An in vitro study carried out by Freire et al. showed that PRGF eye drops significantly increase corneal epithelial cell (HCE) proliferation compared to AS [20]. These results were corroborated in a posterior study in which PRGF eye drops reduced the corneal re-epithelialization time in an in vivo mechanical de-epithelialization model in rabbits regarding AS treatment [21]. A subsequent in vitro study demonstrated that PRGF eye drops are capable of exerting a higher anti-inflammatory effect than AS on ocular surface fibroblasts treated with proinflammatory IL-1β and TNFα [22]. Finally, a recent study showed that PRGF eye drops were able to produce a significant reduction in the number of transforming growth factor (TGF)-β1-induced myofibroblasts in comparison to AS, suggesting that PRGF may promote corneal wound healing regeneration, reducing scar formation [15]. To understand the pathways by which PRGF exerts its antifibrotic potential in comparison to AS, a proteomic study was performed in HK cells differentiated to myofibroblasts, which previously were to be treated with PRGF or AS [23]. This study shows that PRGF treatment inactivated or reduced the activation of several proteins involved in the pathways, whereby TGF-β1 exerts its action to induce the formation of α-actin fibers on human corneal stromal keratocytes (HK), inducing their transformation to myofibroblasts.

It is important to mention that all of the studies described above were carried out with 20% diluted AS eye drops as they are usually used in clinical practice in order to reduce the concentration of TGF-β1 to prevent its potentially harmful effect [12,13]. However, recent groups and studies advocate the use of 100% AS, increasing the concentration of other beneficial factors involved in ocular wound healing like epidermal growth factor (EGF) or fibronectin to achieve better clinical outcomes [24,25].

The purpose of the present study has been to characterize and quantify the protein composition of 100% PRGF in comparison to 100% AS. In addition, the differential protein expression of HK cells after treatment with PRGF or AS was also determined.

## 2. Results

### 2.1. Hematological Characterization of PRGF

PRGF preparations had a mean platelet enrichment of 2.0 ± 0.4-fold (431 ± 133 × 10^3^ platelets/μL) over the platelet concentration in peripheral blood (215 ± 39 × 10^3^ platelets/μL). In addition, minimum levels of leucocytes (0.2 ± 0.1 × 10^3^ leucocytes/μL) were observed in the PRGF preparations. PRGF is classified depending on the type of PRP classification, as is described in Table 1.

### 2.2. Proteomic Characterization of Blood-Derived Products

**Figure 1:**
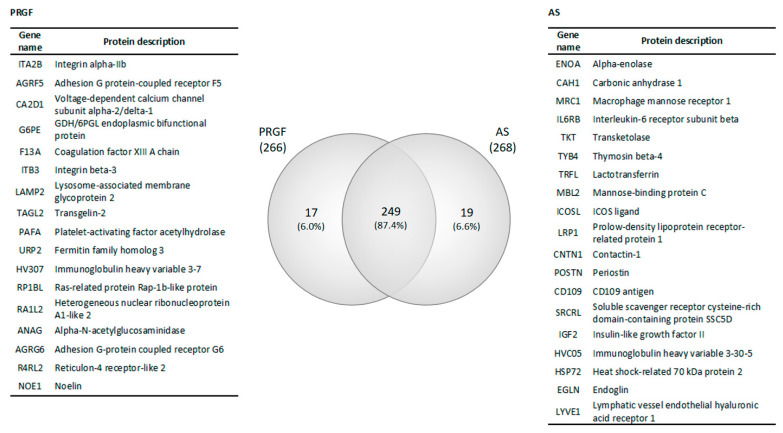
Venn diagram comparison of two blood-derived fractions and the list of proteins specific for PRGF and AS.

Protein samples coming from the two conditions (PRGF and AS) and obtained from the three donors were analyzed independently for differential protein expression. About 285 different proteins were detected in total between both types of formulations. For the complete list of proteins in these formulations and their relative expression see Supplementary File S1. The Venn diagram in Figure 1 summarizes the intersection of the total proteins in both blood-derived products (PRGF and AS). In summary, the number of proteins identified in each formulation was 266 and 268, respectively. The Venn diagram shows that 249 proteins (87.4% of all proteins) were shared by both formulations, while 17 proteins (6.0% of the total) were only identified in the PRGF formulation, and 19 (6.6%) were specific to the AS product. The lists of proteins shared or specific to each formulation are included in Supplementary File S1.

**Figure 2:**
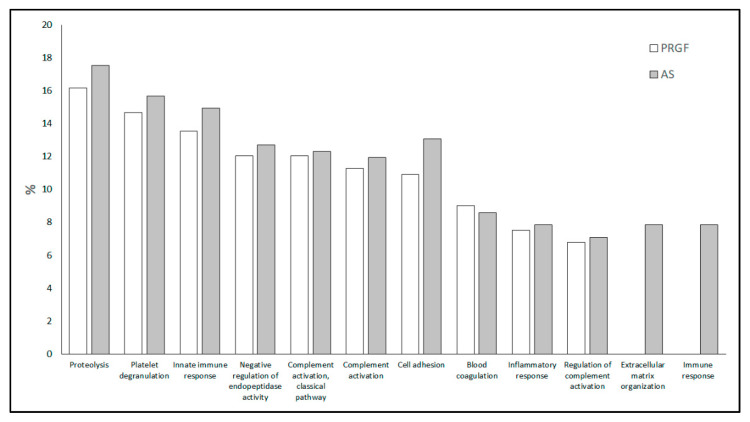
The ten most abundant Gene Ontology terms identified in PRGF and AS formulations, and two of the processes that were only found in AS formulation, which were related to extracellular matrix organization and immune response.

A GO analysis was carried out with the aim of characterizing the functional processes these proteins are involved in. A total of 259 different GO terms were found between the two blood-derived products (PRGF and AS). The lists of all GO terms corresponding to each formulation can be found in Supplementary File S1. Figure 2 shows the twelve most abundant GO terms found between the two formulations (PRGF and AS). The first ten GO terms shared by all formulations may be grouped into 3 main processes: (i) Cellular activity, consisting on proteolysis, negative regulation of endopeptidase activity and cell adhesion GO terms; (ii) Immune response combining the GO terms of innate immune response, complement activation (classical pathway), complement activation, inflammatory response and regulation of complement activation; and (iii) Platelet function, which comprises the GO terms of platelet degranulation and blood coagulation. Although the percentage of differentially expressed proteins involved in all these GOs are similar for every blood-derived product, there are at least two processes for each, such as extracellular matrix organization and immune response, meaning that some proteins were found in AS but not in PRGF.

PRGF and AS protein composition was compared by a relative quantitative proteomics analysis. A total of 257 proteins were quantified with at least two different peptides (Supplementary File S2), of which 13 had a *p* value < 0.05 and a ratio > 1.5 in either direction. These proteins were selected for further analysis (Table 2).

The GO analysis showed that these differentially expressed proteins could be involved in different biological processes (Table 3). However, when IPA analysis was performed to characterize the functional processes in which the differentially expressed proteins are involved, no significant differences were found in any of the processes identified (Supplementary File S3).

### 2.3. Proteomic Characterization of HK Cells Treated with Blood-Derived Products

Protein samples coming from HK cells treated with both conditions (PRGF and AS) obtained from three different donors were analyzed for differential expression. A total of 3236 proteins were quantified, and 352 of them showed statistically significant differences (Supplementary File S4). Further analyses were carried out to study these 352 differentially expressed proteins in HK cells after PRGF and AS treatment. A Gene Ontology (GO) analysis was carried out to initially characterize the functional processes that these significantly differential expressed proteins are involved in, and a total of 166 GO terms were significantly enriched (Supplementary File S4).

**Figure 3:**
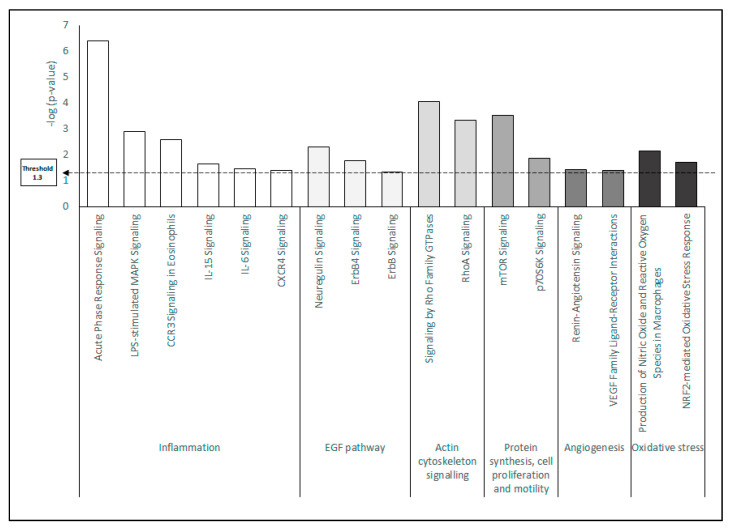
Canonical pathway analysis of the differentially expressed proteins in HK cells after treatment with PRGF or AS. The most significantly enriched canonical pathways (−log *p* values in the y axis) are displayed. The results are clustered in functionally related groups of processes: Inflammation; EGF pathway; Actin cytoskeleton signaling; Protein synthesis, cell proliferation and motility; Angiogenesis; and Oxidative stress.

Ingenuity pathways analysis (IPA) was accomplished for further characterization of the functional processes in which the proteins with significant differential expression were involved. The comparison of protein expression in HK cells treated with PRGF and AS showed that several pathways were significantly deregulated. Figure 3 summarizes the canonical pathways that were most enriched among PRGF vs. AS differentially expressed proteins. This analysis revealed that these canonical pathways were distributed mainly between six relevant biological functions: (A) Inflammation; (B) EGF pathway; (C) Actin cytoskeleton signaling; (D) Protein synthesis, cell proliferation and motility; (E) Angiogenesis; and (F) Oxidative stress. The IPA Z-score, which provides an estimation of the expected activation/inhibition of these pathways based on the expression pattern of the differentially regulated proteins, suggests an increased activity for these functions in AS-mediated response in comparison to PRGF-mediated response (Supplementary File S5). Six of the 17 canonical pathways are related to signaling pathways activated by inflammatory mediators such as Acute Phase Response Signaling, LPS-stimulated MAPK Signaling, CCR3 Signaling in Eosinophils, IL-6 Signaling, IL-15 Signaling and CXCR4 Signaling (Figure 3).

**Figure 4:**
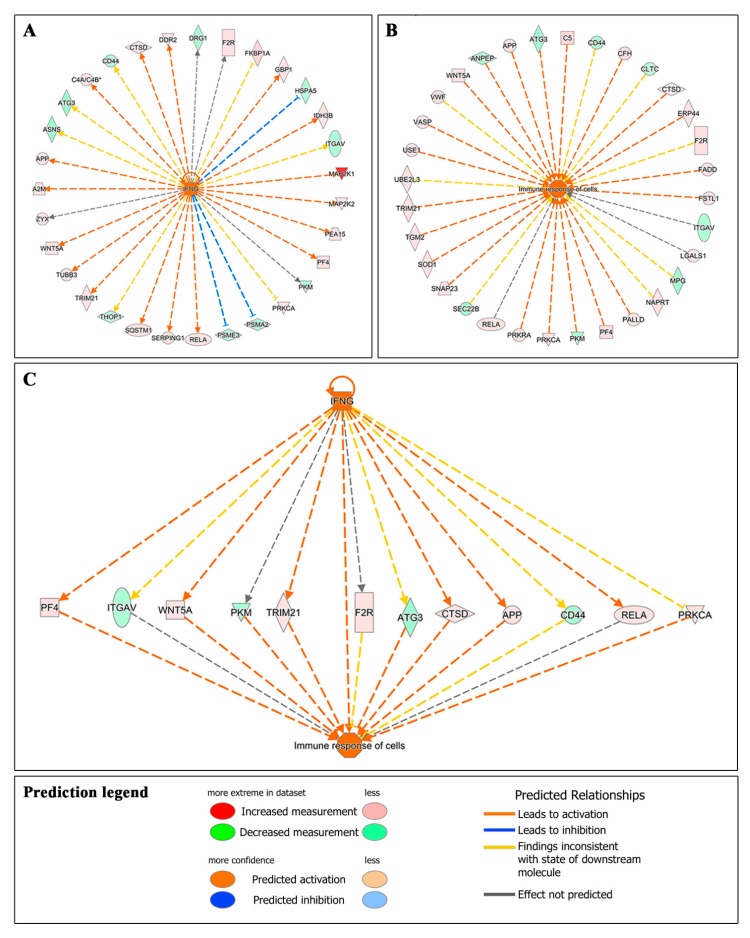
IPA analysis of differentially expressed proteins related to an immune response. (**A**) the IPA upstream regulators analysis suggests that several proteins differentially expressed with AS treatment are related to the activation of HK by IFN-γ. (**B**) IPA downstream analysis also showed that numerous differentially expressed proteins are associated with an immune response of HK cells treated with undiluted AS. (**C**) Finally, IPA upstream/downstream analysis link some differentially expressed proteins with the activation of HK cells by IFN-γ and the induction of an immune response.

The significant association between the differentially expressed proteins in HK cells after AS treatment with the inflammation-related terms is higher than with PRGF, suggesting a great correlation between AS and inflammatory induction. Furthermore, the IPA upstream regulator analysis suggests that several differentially expressed proteins after AS treatment are related to the activation of HK cells by some inflammatory cytokines like IFN-γ (Figure 4A). In addition, the IPA disease and biofunctions analyses showed that several proteins enriched in HK cells treated with AS in comparison to those treated with PRGF are related to the development of a cellular immune response (Figure 4B). Finally, when upstream regulators are related with disease and biofunctions, the IPA analysis showed that some differentially expressed proteins after AS treatment correlate the IFN-γ activation of HK cells with the immune response of these cells (Figure 4C). All the above results demonstrate that AS treatment induce a significantly higher inflammatory response in HK cells when compared to PRGF treatment.

Three additional canonical pathways (Neuregulin Signaling, ErbB4 Signaling, and ErbB Signaling) are related to EGF response (Figure 3). The EGF pathway activation seems to be more highly correlated to AS treatment than to PRGF.

**Figure 5:**
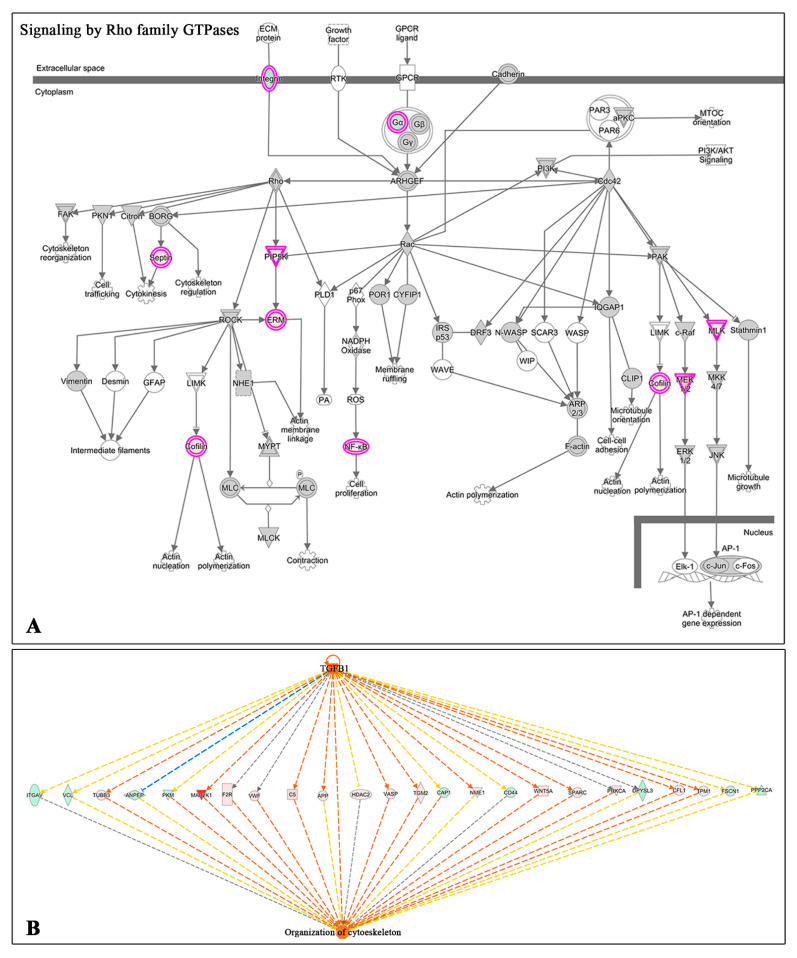
IPA analysis of differentially expressed proteins related to actin cytoskeleton activation. (**A**) Signaling cascade of Rho Family GTPases pathway. Red circles represent over-expression of proteins in HK ells treated with undiluted AS. (**B**) IPA upstream/downstream analysis link some differentially expressed proteins with the activation of HK cells by TGF-β1 and the activation of the cytoskeleton organization.

Two additional canonical pathways, like signaling by Rho Family GTPases and RhoA Signaling, related to actin cytoskeleton signaling, were significantly more activated in HK cells treated with AS compared to PRGF (see Figure 3, Supplementary Figure S1 and Figure 5A). Furthermore, the IPA upstream regulator analysis revealed that some differentially expressed proteins with a significant difference between AS and PRGF treatment were related to TGF-β1 activation (Supplementary Figure S2). In addition, the IPA downstream analyses showed that many of these differentially expressed proteins in HK cells treated with AS were related to the activation of the organization of cytoskeleton (Supplementary Figure S3). Finally, a combination of upstream and downstream pathways showed that some differentially expressed proteins after AS treatment correlate the possible activation of HK cells with TGF-β1 and the activation of the organization of the cytoskeleton (Figure 5B). The significant association between the differentially expressed proteins in HK cells after AS treatment with the cytoskeleton-related terms is higher than with PRGF, suggesting a tight correlation between AS and cytoskeletal functions.

Other important processes related to wound healing, such as angiogenesis and protein synthesis, cell proliferation and motility, were also represented with two canonical pathways, each one like Renin-Angiotensin Signaling and VEGF Family Ligand-Receptor and Interactions mTOR signaling and p70S6K signaling, respectively (Figure 3). In both cases, AS treatment significantly increased the number of differentially expressed proteins in HK cells in comparison to PRGF.

**Figure 6:**
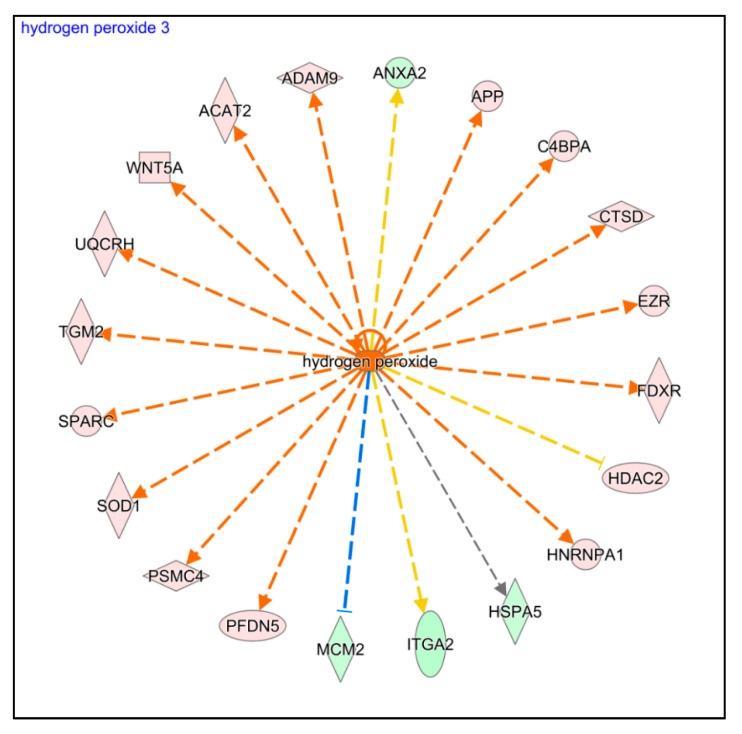
IPA Comparison Analysis predicted hydrogen peroxide as a top upstream regulator for some protein differentially expressed in HK cells after AS treatment.

Finally, two more regulatory pathways related to oxidative stress (Production of Nitric Oxide and Reactive Oxygen Species in Macrophages and NRF2-mediated Oxidative Stress Response) were also significantly activated in AS compared to PRGF (Figure 3 and Supplementary Figure S4). In addition, the IPA upstream analysis revealed that some differentially expressed proteins were related to the activation of HK cells by oxidative stress, similar to the induction of HK cells by the addition of hydrogen peroxide (Figure 6). These results suggest that oxidative stress is significantly increased in HK cells after AS treatment in comparison to PRGF.

## 3. Discussion

Over the past four decades, an increasing number of blood-derived products have been developed to improve tissue regeneration in several ocular surface diseases. There are several protocols and processes that have been established to produce this type of blood derivative, obtaining different products which contain different protein compositions, leading to a wide variety of clinical outcomes for the treatment of the same ocular surface disorders. The first blood-derived product used for the treatment of ocular surface pathologies was autologous serum (AS) diluted at 20%. However, when platelets were found to be one of the most important sources of protein and growth factors with regenerative properties, blood-derived products enriched in platelets were increasing their popularity in their use for the treatment of ocular pathologies due to their higher content in growth factors regarding AS [9].

Apart from this, autologous serum was empirically used from the outset diluted at 20% because the concentration of several growth factors with antiproliferative and pro-fibrotic properties like TGF-β were observed that were 5 times higher in AS than in tears. Since then, autologous serum eye drops were prepared at 20% dilution to prevent the potentially harmful effect [13,33]. However, AS dilution may reduce the concentration of several beneficial factors proven to support proliferation and migration of corneal epithelial cells [13]. Nonetheless, in the last few years, several groups and studies have supported the use of AS at higher concentrations of 50–100% to increase the concentration of growth factors with regenerative potential in touch with the damaged tissue, demonstrating good results in terms of both efficacy and safety [24,34,35].

We used sets of samples (PRGF and AS) from three donors. The first step of this study was to conduct a characterization of blood-derived products (undiluted PRGF and AS), which showed that PRGF contains two-fold higher platelet concentrations than platelets contained in peripheral blood used to obtain AS. Similar results were also observed in a comparative study between PRGF and AS, where the concentration of platelets in PRGF samples was almost twice that of AS [15]. In addition, in the present study, we present a proteomic analysis of the eye drops derived from PRGF in comparison with undiluted AS. Furthermore, the comparative proteomic expression between HK cultured cells treated with PRGF or AS was also determined.

When the proteomic analyses of the different blood-derived products were carried out, the results showed that only 285 proteins were identified between both formulations (PRGF and AS). Of all the proteins identified between both blood-derived formulations, significant differences between PRGF and AS were only found in 13 of them. However, these differences were not related to the activation of any biological pathways. These results suggest that there would be no differences between PRGF and AS. However, in spite of the depletion of the most abundant proteins, the large dynamic range of protein concentrations in blood-derived products, more than 10 orders of magnitude could mask the proteins with a lower concentration in contrast to high abundance proteins [36,37]. Therefore, an analysis of scarce proteins using further proteomic methods to unravel the differences in the protein composition of these two products (PRGF and AS) could be necessary.

In contrast, 3236 differentially expressed proteins were found when the proteome obtained from HK cells treated with PRGF or AS were compared, of which 352 were shown to have statistically significant differences. The analysis of the processes which these proteins are involved in revealed that these processes could be summarized as having six main biological functions: (A) Inflammation; (B) Angiogenesis; (C) Oxidative stress; (D) EGF pathway; (E) Protein synthesis, cell proliferation and motility; and (F) Actin cytoskeleton signaling. All these biological functions were significantly increased in HK cells treated with AS rather than in those cells treated with PRGF.

These results suggest a close relationship between AS-treated cells and the activation of different pathways in HK cells related to an inflammatory response, which was mainly related to Acute Phase Response Signaling, LPS-stimulated MAPK Signaling, CCR3 Signaling in Eosinophils, IL-6 Signaling, IL-15 Signaling and CXCR4 Signaling. These results were confirmed with an IPA analyses, which showed that several proteins, differentially expressed in a statistically significant form in HK cells treated with AS, may be associated with a cellular activation by some inflammatory cytokines such as IFN-γ, which leads to an immune response in the HK cells (see Figure 4). All these pathways associated with an immune response in HK cells after AS treatment could be related to the presence of inflammatory cytokines like IL-1, IL-6, IL-15, TNFα or IFN-γ in AS samples derived from the presence of macrophages and leukocytes during the preparation of this type of blood-derived product [38,39]. The etiopathology of several ocular diseases has an immunological component, or is secondary to systemic inflammatory diseases such as Sjögren’s syndrome, rheumatoid arthritis, diabetes, graft versus host disease, among others [40,41,42]. Therefore, these ocular diseases should be treated with a therapy with a low content in inflammatory cytokines, which us able to regenerate the damaged tissue, exerting at the same time an anti-inflammatory effect. A recent study showed that PRGF exerts a higher anti-inflammatory effect than AS diluted at 20% in an in vitro model of ocular surface fibroblasts treated with proinflammatory IL-1β and TNFα [22]. The present results suggest that undiluted AS could induce a higher inflammatory response in HK cells than PRGF eye drops.

In addition, inflammatory processes in pathologic cornea may stimulate the production of angiogenic factors by different ocular surface cells like epithelial cells or keratocytes. Some of these factors, such as vascular endothelial growth factor (VEGF), have been identified and isolated from the cornea [43]. The cornea is a unique avascular tissue, which gives it the characteristics of transparency and regularity that are essential for maintaining the optical function of the eyes. Many corneal disorders, such as infection, injury, and autoimmune reactions, lead to corneal angiogenesis. Corneal invasion by vessels induces its opacification, reducing patient visibility. Therefore, it is essential to reduce the possibility of inducing an angiogenic effect from drugs used to treat ocular surface pathologies. The present study shows that AS treatment induces a higher activation of proteins related to Renin-Angiotensin Signaling and VEGF Family Ligand-Receptor pathways than PRGF, suggesting that AS treatment may induce a significant higher activation of an angiogenic response in HK cells rather than PRGF eye drops.

On the other hand, several studies have demonstrated that inflammation has a strong association with oxidative stress [44]. Oxidative stress is characterized by the production of reactive oxygen species (ROS), like the superoxide anions, hydrogen peroxide, and hydroxyl radicals, which are related to cell damage inducing lipid peroxidation of membranes, oxidative changes in proteins, and oxidative damage to DNA [45]. An inflammatory process could induce an increase in ROS levels due to fueled oxygen consumption or reduced antioxidative defense in the affected tissue [46]. Several ocular disorders, like corneal inflammation, dry eye disease, keratoconus, and Fuchs’ endothelial dystrophy, are associated with oxidative stress [47,48]. Several antioxidant treatments have been proposed to reduce the inflammatory reaction in several ocular diseases trying to induce ocular tissue healing [47,49,50]. Recent studies showed that PRGF treatment reduced the cytotoxic effects induced in retinal pigment epithelial cells exposed to an oxidative stress environment modulating the antioxidant pathways [51,52]. The present study shows that undiluted AS treatment induces an activation of oxidative stress pathways in HK cells similar to induction by the addition of hydrogen peroxide. The proteins related to oxidative stress were differentially expressed in a statistically significant form in HK cells treated with AS in comparison to those treated with PRGF, as is shown in Figure 6.

In addition, the present results revealed that proteins which are more abundant in AS-treated cells were clustered into an additional canonical pathway group related to EGF pathway activation. EGF, through binding to the EGF receptor (EGF-R), stimulates the proliferation of corneal epithelial and endothelial cells and accelerates epithelial wound healing [53,54]. Furthermore, EGF promotes cell motility through its receptor phosphorylation, leading to an actin cytoskeletal rearrangement [55]. However, in the case of keratocytes, EGF induces cell differentiation to myofibroblasts through the stimulation of the EGF-R signaling pathway [56]. Myofibroblasts are responsible for wound contraction and extracellular matrix deposition and organization during injury repair. However, the continued presence of myofibroblasts after wound healing has been found to be the primary biological episode responsible for the development of scarring tissue [57]. Therefore, the present results suggest that the activation of EGF pathways in HK cells after AS treatment may promote scar tissue formation compromising corneal transparency.

Furthermore, AS-treated cells reveal a greater association with processes such as protein synthesis, proliferation, and cellular motility, mainly related to mTOR (mammalian target of rapamycin) signaling and p70S6K (Ribosomal protein S6 kinase beta-1) signaling. The p70S6K is a downstream target of mTOR signaling and the mTOR is a serine/threonine protein kinase that has been found to affect many cellular functions, including cell growth, proliferation, and metabolism [58]. In addition, in a recent study of a rabbit alkali burn model, it was shown that the inhibition of the mTOR pathway promoted the autophagy and inhibited the proliferation, invasion, and migration of corneal stromal cells, promoting corneal wound healing [59]. Furthermore, it has been shown that mTOR signaling may induce scarring, neovascularization, and inflammation in the cornea [60,61]. An additional study showed that TGF-β activated the mTOR pathway in corneal stromal fibroblasts, and that rapamycin (a mTOR inhibitor) inhibited corneal stromal fibroblasts proliferation and modulated their transformation into myofibroblasts [62]. Hence, the mTOR signaling pathway activation after undiluted AS treatment could induce a higher tissue fibrosis than PRGF. In the same way, significant pathway activation associated with actin cytoskeleton signaling (Rho Family GTPases and RhoA Signaling) has been showed in HK cells treated with undiluted AS in comparison to PRGF. Rho family GTPases control diverse signal transduction pathways, one of the main functions of which is to control the actin cytoskeleton. Members of the Rho family GTPases include RhoA, -B, and -C, Rac1 and -2, and Cdc42. RhoA regulates actin polymerization, inducing the formation of stress fibers and the assembly of focal adhesion complex [63]. Several growth factors, like TGF-β and basic fibroblast growth factor (FGF)-2, induce the activation of Rho signaling pathways; however, TGF-β seems to be the main factor which activates these pathways and therefore is the main inductor to keratocyte differentiation to myofibroblasts, leading to the expression of α-SMA [64,65]. In the present study, the results observed after IPA analyses showed that a large number of proteins differentially expressed in a statistically significant manner in HK cells treated with AS were related to the activation of the organization of cytoskeleton similar to the stimulation of HK cells by TGF-β1, as is shown in Figure 5. Our results come along with previous proteomic study in HK myodifferentiated cells after TGF-β1 incubation and treatment with PRGF or 20% AS [23]. This study showed that PRGF treatment inactivated or reduced the activation of several proteins involved in the pathways whereby TGF-β1 exerts its action to induce the formation of α-actin fibers on HK cells, inducing their transformation to myofibroblasts. However, AS treatment was not able to reduce TGF-β1 action in myodifferentiated HK cells. According to this, previous studies have also demonstrated that PRGF exerts an anti-fibrotic effect by reducing the transformation of TGF-β1-treated stromal fibroblasts to myofibroblasts [66], minimizing scar formation while improving corneal tissue regeneration [67]. Furthermore, it has been shown that this anti-fibrotic effect of PRGF is significantly higher than AS diluted at 20% in HK cells differentiated to myofibroblasts by TGF-β1 [15]. The higher content of fibroblast growth factor (FGF) in PRGF formulations rather than in AS may be a possible mechanism by which PRGF induces less activation of the actin cytoskeleton pathways. Some studies have demonstrated that FGF-1 and -2 promote the fibroblast phenotype and reverse the myofibroblast phenotype [68].

The present study suggests that undiluted AS induces the activation of different pathways in HK cells related to an inflammatory, angiogenic, oxidative stress and scarring response in comparison to PRGF. Thus, PRGF could be a better alternative than AS for the treatment of ocular surface disorders. However, blood-derived products are composed of a wide variety of proteins and growth factors; hence, further studies are needed to unravel the proteins involved in the mechanisms underlying the differentially regulated pathways between PRGF and AS.

## 4. Materials and Methods

### 4.1. PRGF and Autologous Serum (AS) Preparations

**Figure 7:**
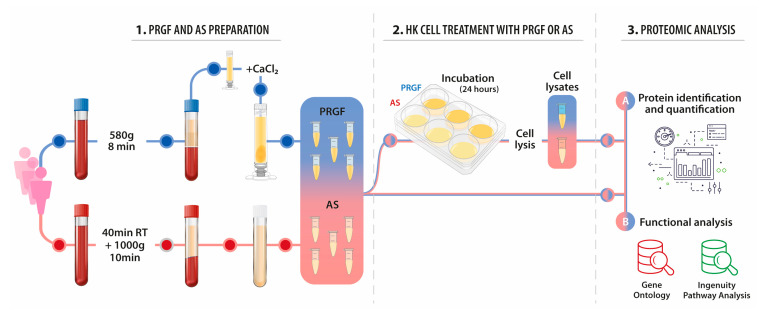
The study design is shown schematically. First, the two blood derivatives, PRGF and AS, were obtained. On the one hand, HK cells were incubated with these formulations to assess their response to each blood derived product by proteomic techniques. On the other hand, both PRGF and AS preparations were characterized by the same proteomic techniques.

Blood from three healthy male donors was drawn off after obtaining informed consent into 9 mL tubes with 3.8% (w/v) sodium citrate or in serum collection tubes (Z Serum Clot activator, Vacuette, GmbH, Kremsmünster, Austria) for PRGF and AS preparation respectively (Figure 7). The study was performed following the principles of the Declaration of Helsinki. Blood sample for PRGF was centrifuged at 580× *g* for 8 min at room temperature in an Endoret System centrifuge (BTI Biotechnology Institute, S.L., Vitoria, Álava, Spain); the whole plasma column was collected using Endoret ophthalmology kit (BTI Biotechnology Institute, S.L., Vitoria, Álava, Spain) avoiding the buffy coat collection. Platelets and leukocytes counts were performed with a hematology analyzer (Pentra ES 60, Horiba ABX SAS, Montpelier, France). The whole plasma was activated with 10% calcium chloride at a rate of 20 μL per milliliter of PRGF, and the obtained PRGF supernatants were filtered, aliquoted and stored at −80 °C (the undiluted PRGF was termed PRGF). Blood sample for autologous serum preparation was allowed to clot at room temperature for 40 min and then it was centrifuged for 10 min at 1000× *g*; after that, serum was collected and filtered by 0.22 μm PVDF filters, aliquoted and stored at −80 °C until use (the undiluted AS was termed AS).

### 4.2. Cells

Cells involved in assays were human corneal stromal keratocytes (HK) (ScienCell Research Laboratories, San Diego, CA, USA) that were cultured according to the manufacturer’s instructions. Briefly, cells were maintained in culture until confluence in Fibroblast medium supplemented with Fibroblast Growth Supplement (FGS), 2% fetal bovine serum (FBS) and antibiotics (penicillin/streptomycin) (ScienCell Research Laboratories, San Diego, CA, USA) and then were detached with animal origin-free trypsin-like enzyme (TrypLE Select, Gibco-Invitrogen, Grand Island, NY, USA). Cell viability was assessed by trypan blue dye exclusion.

HK cells were seeded in a 6-well plate at a density of 50,000 cells per cm^2^ in serum-free medium supplemented with 20% (*v/v*) of the different treatment samples (PRGF and AS) obtained from the three donors (Figure 7). The HK cells were incubated with each treatment for 24 h. Then, culture media were discarded and the wells were rinsed with PBS. In order to obtain the proteins from cells, 400 μL of cell lysis buffer consisting of 7 M urea, 2 M thiourea, 4% CHAPS was added to each well. Samples were incubated for 30 min at room temperature under agitation and digested following the filter-aided FASP protocol described by Wisniewski et al. [69] with minor modifications. Trypsin was added to a trypsin:protein ratio of 1:10, and the mixture was incubated overnight at 37 °C, dried out in a RVC2 25 speedvac concentrator (Christ), and resuspended in 0.1% FA.

### 4.3. Proteomic Analysis

Samples were submitted to LC-MS label-free analysis using a novel hybrid trapped ion mobility spectrometry–quadrupole time of flight mass spectrometer (timsTOF Pro with PASEF, Bruker Daltonics, Billerica, MA, USA) coupled online to a nanoElute liquid chromatograph (Bruker. Sample (200 ng) was directly loaded in a 15 cm Bruker nanelute FIFTEEN C18 analytical column (Bruker) and resolved at 400 nL/min with a 30 min gradient. Column was heated to 50 °C using an oven.

Protein identification and quantification was carried out using MaxQuant software using default settings [70]. Searches were carried out against a database consisting of human protein entries (Uniprot/Swissprot), with precursor and fragment tolerances of 20ppm and 0.05 Da. Only proteins identified with at least two peptides at FDR < 1% were considered for further analysis. Data (LFQ intensities) were loaded onto Perseus platform [71] and further processed (log2 transformation, imputation) before statistical analysis (Student’s *t*-test).

### 4.4. Functional Analysis

Gene Ontology (GO) enrichment analysis was carried out using the DAVID online tool (http://david.abcc.ncifcrf.gov/summary.jsp accessed 7 October 2020) [72,73]. DAVID is a GO Term annotation and enrichment analysis tool used to highlight the most relevant GO terms associated with a given gene list. A Fisher’s Exact test is used in order to determine whether the proportion of genes considered into certain GO term or categories differ significantly between the dataset and the background. Biological Process (BP), Molecular Function (MF) and Cellular Component (CC) categories were assessed, and only GO Terms enriched with a *p* value < 0.05 were considered for comparison and discussion.

Ingenuity Pathway Analysis (IPA, QIAGEN Redwood City, www.qiagen.com/ingenuity accessed on 7 October 2020) was used for the functional analysis of the proteins identified. The calculated *p*-values determine the probability that the association between proteins in the dataset and a given canonical pathway, functional network or upstream regulator is explained by chance alone, based on a Fisher’s exact test (*p* value < 0.05 considered as significant). Activation z-score represents the bias in gene regulation that predicts whether the upstream regulator exists in an activated (positive values) or inactivated (negative values) state, based on a knowledge of the relationship between the effectors and their target molecules.

## 5. Conclusions

Although further studies are needed to find the possible proteomic differences between PRGF and AS due to the large dynamic range of protein concentrations in these types of blood-derived products, the present study showed that PRGF and AS induce a significantly difference response in HK cells. Undiluted AS induces the activation of different pathways in HK cells related to an inflammatory, angiogenic, oxidative stress and scarring response in comparison to PRGF. These results suggest that PRGF could be a better alternative to AS for the treatment of ocular surface disorders.

## Figures and Tables

**Table 1:** PRGF-Endoret system (BTI Biotechnology Institute) classification.

Classification	Reference	PRGF-Endoret
MISHRA	[26]	4 (B)
PAW	[27]	P2-x-Bβ
PLRA	[28]	P0.5 L-R-A+
DEPA	[29]	CCA
ISTH	[30]	PRP IIA1
MARSPILL	[31]	M A+ RBC-P Sp1 PL2-3 Lc-P A-
Consensus Experts	[32]	24-00-11

**Table 2:** Statistically significant de-regulated proteins between AS vs. PRGF eye drops.

Protein Accession Number	Gene Name	Protein Description	Fold Change	*p*-Value
Q9UK55	ZPI	Protein Z-dependent protease inhibitor	1.2	0.0001
P68871	HBB	Hemoglobin subunit beta	31.4	0.0098
P02788	TRFL	Lactotransferrin	2.8	0.0156
P03952	KLKB1	Plasma kallikrein	1.1	0.0168
P14151	LYAM1	L-selectin	1.1	0.0192
P01024	CO3	Complement C3	1.0	0.0193
P08603	CFAH	Complement factor H	1.1	0.0253
P62328	TYB4	Thymosin beta-4	3.2	0.0255
P00915	CAH1	Carbonic anhydrase 1	13.2	0.0301
Q92496	FHR4	Complement factor H-related protein 4	1.7	0.0329
P08709	FA7	Coagulation factor VII	1.4	0.0428
P22105	TENX	Tenascin-X	0.9	0.0474
P01860	IGHG3	Immunoglobulin heavy constant gamma 3	1.2	0.0491

**Table 3:** Gene Ontology analysis of de-regulated proteins with statistically significant differences between AS vs. PRGF.

GO Term	GO Definition	Genes	%	*p*-Value
0006956	Complement activation	P08603, P01860, P01024	23.1	0.0017
0006508	Proteolysis	P03952, P02788, P01860, P01024	30.8	0.0047
0007596	Blood coagulation	P68871, P08709, Q9UK55	23.1	0.0073
0006957	Complement activation, alternative pathway	P08603, P01024	15.4	0.0093
0030449	Regulation of complement activation	P08603, P01024	15.4	0.0212
0001895	Retina homeostasis	P02788, P01860	15.4	0.0282
0015701	Bicarbonate transport	P00915, P68871	15.4	0.0310
0006958	Complement activation, classical pathway	P01860, P01024	15.4	0.068
0010951	Negative regulation of endopeptidase activity	Q9UK55, P01024	15.4	0.0831
0030036	Actin cytoskeleton organization	P62328, P22105	15.4	0.0891

## Data Availability

All the obtained data used to support the findings of this study are available from the corresponding author upon reasonable request.

## Supplementary Materials

The following are available online at https://www.mdpi.com/article/10.3390/ijms222212176/s1.

## References

[1] Lemp M.A. (2008). Management of dry eye disease. Am. J. Manag. Care.

[2] Blomquist P.H. (2011). Ocular complications of systemic medications. Am. J. Med. Sci..

[3] Pan Q., Angelina A., Marrone M., Stark W.J., Akpek E.K. (2017). Autologous serum eye drops for dry eye. Cochrane Database Syst. Rev..

[4] Schultz C. (2014). Safety and efficacy of cyclosporine in the treatment of chronic dry eye. Ophthalmol. Eye Dis..

[5] Inatomi T., Nakamura T., Kojyo M., Koizumi N., Sotozono C., Kinoshita S. (2006). Ocular surface reconstruction with combination of cultivated autologous oral mucosal epithelial transplantation and penetrating keratoplasty. Am. J. Ophthalmol..

[6] Bonini S., Lambiase A., Rama P., Caprioglio G., Aloe L. (2000). Topical treatment with nerve growth factor for neurotrophic keratitis. Ophthalmology.

[7] Manni L., Rocco M.L., Bianchi P., Soligo M., Guaragna M., Barbaro S.P., Aloe L. (2013). Nerve growth factor: Basic studies and possible therapeutic applications. Growth Factors.

[8] Márquez E.B., De Ortueta D., Royo S.B., Martínez-Carpio P.A. (2011). Epidermal growth factor receptor in corneal damage: Update and new insights from recent reports. Cutan. Ocul. Toxicol..

[9] Anitua E., Muruzabal F., Tayebba A., Riestra A., Perez V.L., Merayo-Lloves J., Orive G. (2015). Autologous serum and plasma rich in growth factors in ophthalmology: Preclinical and clinical studies. Acta Ophthalmol..

[10] Riestra A.C., Alonso-Herreros J.M., Merayo-Lloves J. (2016). Platelet rich plasma in ocular surface. Arch. Soc. Esp. Oftalmol..

[11] Pan Q., Angelina A., Zambrano A., Marrone M., Stark W.J., Heflin T., Tang L., Akpek E.K. (2013). Autologous serum eye drops for dry eye. Cochrane Database Syst. Rev..

[12] Giannaccare G., Versura P., Buzzi M., Primavera L., Pellegrini M., Campos E.C. (2017). Blood derived eye drops for the treatment of cornea and ocular surface diseases. Transfus. Apher. Sci..

[13] Geerling G., Maclennan S., Hartwig D. (2004). Autologous serum eye drops for ocular surface disorders. Br. J. Ophthalmol..

[14] Anitua E. (1999). Plasma rich in growth factors: Preliminary results of use in the preparation of future sites for implants. Int. J. Oral Maxillofac. Implant.

[15] Anitua E., de la Fuente M., Muruzabal F., Riestra A., Merayo-Lloves J., Orive G. (2015). Plasma rich in growth factors (PRGF) eye drops stimulates scarless regeneration compared to autologous serum in the ocular surface stromal fibroblasts. Exp. Eye Res..

[16] Anitua E., Zalduendo M.M., Alkhraisat M.H., Orive G. (2013). Release kinetics of platelet-derived and plasma-derived growth factors from autologous plasma rich in growth factors. Ann. Anat..

[17] Sanchez-Avila R.M., Merayo-Lloves J., Riestra A.C., Fernandez-Vega Cueto L., Anitua E., Begoña L., Muruzabal F., Orive G. (2018). Treatment of patients with neurotrophic keratitis stages 2 and 3 with plasma rich in growth factors (PRGF-Endoret) eye-drops. Int. Ophthalmol..

[18] Merayo-Lloves J., Sanchez-Avila R.M., Riestra A.C., Anitua E., Begoña L., Orive G., Fernandez-Vega L. (2016). Safety and Efficacy of Autologous Plasma Rich in Growth Factors Eye Drops for the Treatment of Evaporative Dry Eye. Ophthalmic Res..

[19] Sanchez-Avila R.M., Merayo-Lloves J., Muruzabal F., Orive G., Anitua E. (2020). Plasma rich in growth factors for the treatment of dry eye from patients with graft versus host diseases. Eur. J. Ophthalmol..

[20] Freire V., Andollo N., Etxebarria J., Durán J.A., Morales M.C. (2012). In vitro effects of three blood derivatives on human corneal epithelial cells. Investig. Ophthalmol. Vis. Sci..

[21] Freire V., Andollo N., Etxebarria J., Hernáez-Moya R., Durán J.A., Morales M.C. (2014). Corneal wound healing promoted by 3 blood derivatives: An in vitro and in vivo comparative study. Cornea.

[22] Anitua E., Muruzabal F., de la Fuente M., Riestra A., Merayo-Lloves J., Orive G. (2016). PRGF exerts more potent proliferative and anti-inflammatory effects than autologous serum on a cell culture inflammatory model. Exp. Eye Res..

[23] Anitua E., de la Fuente M., Muruzabal F., Sánchez-Ávila R.M., Merayo-Lloves J., Azkargorta M., Elortza F., Orive G. (2018). Differential profile of protein expression on human keratocytes treated with autologous serum and plasma rich in growth factors (PRGF). PLoS ONE.

[24] Jover Botella A., Márquez Peiró J.F., Márques K., Monts Cambero N., Selva Otaolaurruchi J. (2011). Effectiveness of 100% autologous serum drops in ocular surface disorders. Farm. Hosp..

[25] Lekhanont K., Jongkhajornpong P., Anothaisintawee T., Chuckpaiwong V. (2016). Undiluted Serum Eye Drops for the Treatment of Persistent Corneal Epitheilal Defects. Sci. Rep..

[26] Mishra A., Harmon K., Woodall J., Vieira A. (2012). Sports medicine applications of platelet rich plasma. Curr. Pharm. Biotechnol..

[27] DeLong J.M., Russell R.P., Mazzocca A.D. (2012). Platelet-rich plasma: The PAW classification system. Arthroscopy.

[28] Mautner K., Malanga G.A., Smith J., Shiple B., Ibrahim V., Sampson S., Bowen J.E. (2015). A call for a standard classification system for future biologic research: The rationale for new PRP nomenclature. PM&R.

[29] Magalon J., Chateau A.L., Bertrand B., Louis M.L., Silvestre A., Giraudo L., Veran J., Sabatier F. (2016). DEPA classification: A proposal for standardising PRP use and a retrospective application of available devices. BMJ Open Sport Exerc. Med..

[30] Harrison P., Subcommittee on Platelet P. (2018). The use of platelets in regenerative medicine and proposal for a new classification system: Guidance from the SSC of the ISTH. J. Thromb. Haemost..

[31] Lana J., Purita J., Paulus C., Huber S.C., Rodrigues B.L., Rodrigues A.A., Santana M.H., Madureira J.L., Malheiros Luzo A.C., Belangero W.D. (2017). Contributions for classification of platelet rich plasma—Proposal of a new classification: MARSPILL. Regen. Med..

[32] Kon E., Di Matteo B., Delgado D., Cole B.J., Dorotei A., Dragoo J.L., Filardo G., Fortier L.A., Giuffrida A., Jo C.H. (2020). Platelet-rich plasma for the treatment of knee osteoarthritis: An expert opinion and proposal for a novel classification and coding system. Expert Opin. Biol. Ther..

[33] Imanishi J., Kamiyama K., Iguchi I., Kita M., Sotozono C., Kinoshita S. (2000). Growth factors: Importance in wound healing and maintenance of transparency of the cornea. Prog. Retin. Eye Res..

[34] Noble B.A., Loh R.S., MacLennan S., Pesudovs K., Reynolds A., Bridges L.R., Burr J., Stewart O., Quereshi S. (2004). Comparison of autologous serum eye drops with conventional therapy in a randomised controlled crossover trial for ocular surface disease. Br. J. Ophthalmol..

[35] Jeng B.H., Dupps W.J. (2009). Autologous serum 50% eyedrops in the treatment of persistent corneal epithelial defects. Cornea.

[36] Anderson N.L., Anderson N.G. (2002). The human plasma proteome: History, character, and diagnostic prospects. Mol. Cell Proteom..

[37] Anderson N.L., Polanski M., Pieper R., Gatlin T., Tirumalai R.S., Conrads T.P., Veenstra T.D., Adkins J.N., Pounds J.G., Fagan R. (2004). The human plasma proteome: A nonredundant list developed by combination of four separate sources. Mol. Cell Proteom..

[38] Ma I.H., Chen L.W., Tu W.H., Lu C.J., Huang C.J., Chen W.L. (2017). Serum components and clinical efficacies of autologous serum eye drops in dry eye patients with active and inactive Sjogren syndrome. Taiwan J. Ophthalmol..

[39] Stenwall P.A., Bergström M., Seiron P., Sellberg F., Olsson T., Knutson F., Berglund D. (2015). Improving the anti-inflammatory effect of serum eye drops using allogeneic serum permissive for regulatory T cell induction. Acta Ophthalmol..

[40] Read R.W. (2004). Clinical mini-review: Systemic lupus erythematosus and the eye. Ocul. Immunol. Inflamm..

[41] Stern M.E., Schaumburg C.S., Siemasko K.F., Gao J., Wheeler L.A., Grupe D.A., De Paiva C.S., Calder V.L., Calonge M., Niederkorn J.Y. (2012). Autoantibodies contribute to the immunopathogenesis of experimental dry eye disease. Investig. Ophthalmol. Vis. Sci..

[42] Tabbara K.F., Al-Ghamdi A., Al-Mohareb F., Ayas M., Chaudhri N., Al-Sharif F., Al-Zahrani H., Mohammed S.Y., Nassar A., Aljurf M. (2009). Ocular findings after allogeneic hematopoietic stem cell transplantation. Ophthalmology.

[43] Gan L., Fagerholm P., Palmblad J. (2004). Vascular endothelial growth factor (VEGF) and its receptor VEGFR-2 in the regulation of corneal neovascularization and wound healing. Acta Ophthalmol. Scand..

[44] Yadav U.C., Kalariya N.M., Ramana K.V. (2011). Emerging role of antioxidants in the protection of uveitis complications. Curr. Med. Chem..

[45] Cejka C., Cejkova J. (2015). Oxidative stress to the cornea, changes in corneal optical properties, and advances in treatment of corneal oxidative injuries. Oxid. Med. Cell Longev..

[46] Ishimoto S., Wu G.S., Hayashi S., Zhang J., Rao N.A. (1996). Free radical tissue damages in the anterior segment of the eye in experimental autoimmune uveitis. Investig. Ophthalmol. Vis. Sci..

[47] Augustin A.J., Spitznas M., Kaviani N., Meller D., Koch F.H., Grus F., Göbbels M.J. (1995). Oxidative reactions in the tear fluid of patients suffering from dry eyes. Graefes Arch. Clin. Exp. Ophthalmol..

[48] Buddi R., Lin B., Atilano S.R., Zorapapel N.C., Kenney M.C., Brown D.J. (2002). Evidence of oxidative stress in human corneal diseases. J. Histochem. Cytochem..

[49] Alio J.L., Ayala M.J., Mulet M.E., Artola A., Ruiz J.M., Bellot J. (1995). Antioxidant therapy in the treatment of experimental acute corneal inflammation. Ophthalmic Res..

[50] Cejková J., Ardan T., Cejka C., Luyckx J. (2011). Favorable effects of trehalose on the development of UVB-mediated antioxidant/pro-oxidant imbalance in the corneal epithelium, proinflammatory cytokine and matrix metalloproteinase induction, and heat shock protein 70 expression. Graefes Arch. Clin. Exp. Ophthalmol..

[51] Anitua E., de la Fuente M., Del Olmo-Aguado S., Suarez-Barrio C., Merayo-Lloves J., Muruzabal F. (2020). Plasma rich in growth factors reduces blue light-induced oxidative damage on retinal pigment epithelial cells and restores their homeostasis by modulating vascular endothelial growth factor and pigment epithelium-derived factor expression. Clin. Exp. Ophthalmol..

[52] Suarez-Barrio C., Del Olmo-Aguado S., Garcia-Perez E., de la Fuente M., Muruzabal F., Anitua E., Baamonde-Arbaiza B., Fernandez-Vega-Cueto L., Fernandez-Vega L., Merayo-Lloves J. (2020). Antioxidant Role of PRGF on RPE Cells after Blue Light Insult as a Therapy for Neurodegenerative Diseases. Int. J. Mol. Sci..

[53] Kitazawa T., Kinoshita S., Fujita K., Araki K., Watanabe H., Ohashi Y., Manabe R. (1990). The mechanism of accelerated corneal epithelial healing by human epidermal growth factor. Investig. Ophthalmol. Vis. Sci..

[54] Zieske J.D., Takahashi H., Hutcheon A.E., Dalbone A.C. (2000). Activation of epidermal growth factor receptor during corneal epithelial migration. Investig. Ophthalmol. Vis. Sci..

[55] Maldonado B.A., Furcht L.T. (1995). Epidermal growth factor stimulates integrin-mediated cell migration of cultured human corneal epithelial cells on fibronectin and arginine-glycine-aspartic acid peptide. Investig. Ophthalmol. Vis. Sci..

[56] He J., Bazan H.E. (2008). Epidermal growth factor synergism with TGF-beta1 via PI-3 kinase activity in corneal keratocyte differentiation. Investig. Ophthalmol. Vis. Sci..

[57] Netto M.V., Mohan R.R., Sinha S., Sharma A., Dupps W., Wilson S.E. (2006). Stromal haze, myofibroblasts, and surface irregularity after PRK. Exp. Eye Res..

[58] Zoncu R., Efeyan A., Sabatini D.M. (2011). mTOR: From growth signal integration to cancer, diabetes and ageing. Nat. Rev. Mol. Cell Biol..

[59] Wang Y., Gao G., Wu Y., Wang Y., Wu X., Zhou Q. (2020). S100A4 Silencing Facilitates Corneal Wound Healing after Alkali Burns by Promoting Autophagy via Blocking the PI3K/Akt/mTOR Signaling Pathway. Investig. Ophthalmol. Vis. Sci..

[60] Lee K.S., Ko D.A., Kim E.S., Kim M.J., Tchah H., Kim J.Y. (2012). Bevacizumab and rapamycin can decrease corneal opacity and apoptotic keratocyte number following photorefractive keratectomy. Investig. Ophthalmol. Vis. Sci..

[61] Shin Y.J., Hyon J.Y., Choi W.S., Yi K., Chung E.S., Chung T.Y., Wee W.R. (2013). Chemical injury-induced corneal opacity and neovascularization reduced by rapamycin via TGF-β1/ERK pathways regulation. Investig. Ophthalmol. Vis. Sci..

[62] Milani B.Y., Milani F.Y., Park D.W., Namavari A., Shah J., Amirjamshidi H., Ying H., Djalilian A.R. (2013). Rapamycin inhibits the production of myofibroblasts and reduces corneal scarring after photorefractive keratectomy. Investig. Ophthalmol. Vis. Sci..

[63] Kaibuchi K., Kuroda S., Amano M. (1999). Regulation of the cytoskeleton and cell adhesion by the Rho family GTPases in mammalian cells. Annu. Rev. Biochem..

[64] Jester J.V., Barry-Lane P.A., Cavanagh H.D., Petroll W.M. (1996). Induction of alpha-smooth muscle actin expression and myofibroblast transformation in cultured corneal keratocytes. Cornea.

[65] Chen J., Guerriero E., Sado Y., SundarRaj N. (2009). Rho-Mediated Regulation of TGF-β1– and FGF-2–Induced Activation of Corneal Stromal Keratocytes. Investig. Ophthalmol. Vis. Sci..

[66] Anitua E., Sanchez M., Merayo-Lloves J., De la Fuente M., Muruzabal F., Orive G. (2011). Plasma rich in growth factors (PRGF-Endoret) stimulates proliferation and migration of primary keratocytes and conjunctival fibroblasts and inhibits and reverts TGF-beta1-Induced myodifferentiation. Investig. Ophthalmol. Vis. Sci..

[67] Anitua E., Muruzabal F., Alcalde I., Merayo-Lloves J., Orive G. (2013). Plasma rich in growth factors (PRGF-Endoret) stimulates corneal wound healing and reduces haze formation after PRK surgery. Exp. Eye Res..

[68] Maltseva O., Folger P., Zekaria D., Petridou S., Masur S.K. (2001). Fibroblast growth factor reversal of the corneal myofibroblast phenotype. Investig. Ophthalmol. Vis. Sci..

[69] Wiśniewski J.R., Zougman A., Nagaraj N., Mann M. (2009). Universal sample preparation method for proteome analysis. Nat. Methods.

[70] Cox J., Mann M. (2008). MaxQuant enables high peptide identification rates, individualized p.p.b.-range mass accuracies and proteome-wide protein quantification. Nat. Biotechnol..

[71] Tyanova S., Temu T., Sinitcyn P., Carlson A., Hein M.Y., Geiger T., Mann M., Cox J. (2016). The Perseus computational platform for comprehensive analysis of (prote)omics data. Nat. Methods.

[72] Huang D.W., Sherman B.T., Lempicki R.A. (2009). Bioinformatics enrichment tools: Paths toward the comprehensive functional analysis of large gene lists. Nucleic Acids Res..

[73] Huang D.W., Sherman B.T., Lempicki R.A. (2009). Systematic and integrative analysis of large gene lists using DAVID bioinformatics resources. Nat. Protoc..

