# Supplementary figures and images for: Proteomic Characterization of Plasma Rich in Growth Factors and Undiluted Autologous Serum
Source: Int J Mol Sci. 2021 Nov 10;22(22):12176. doi: 10.3390/ijms222212176 (PMC8618701; doi:10.3390/ijms222212176)

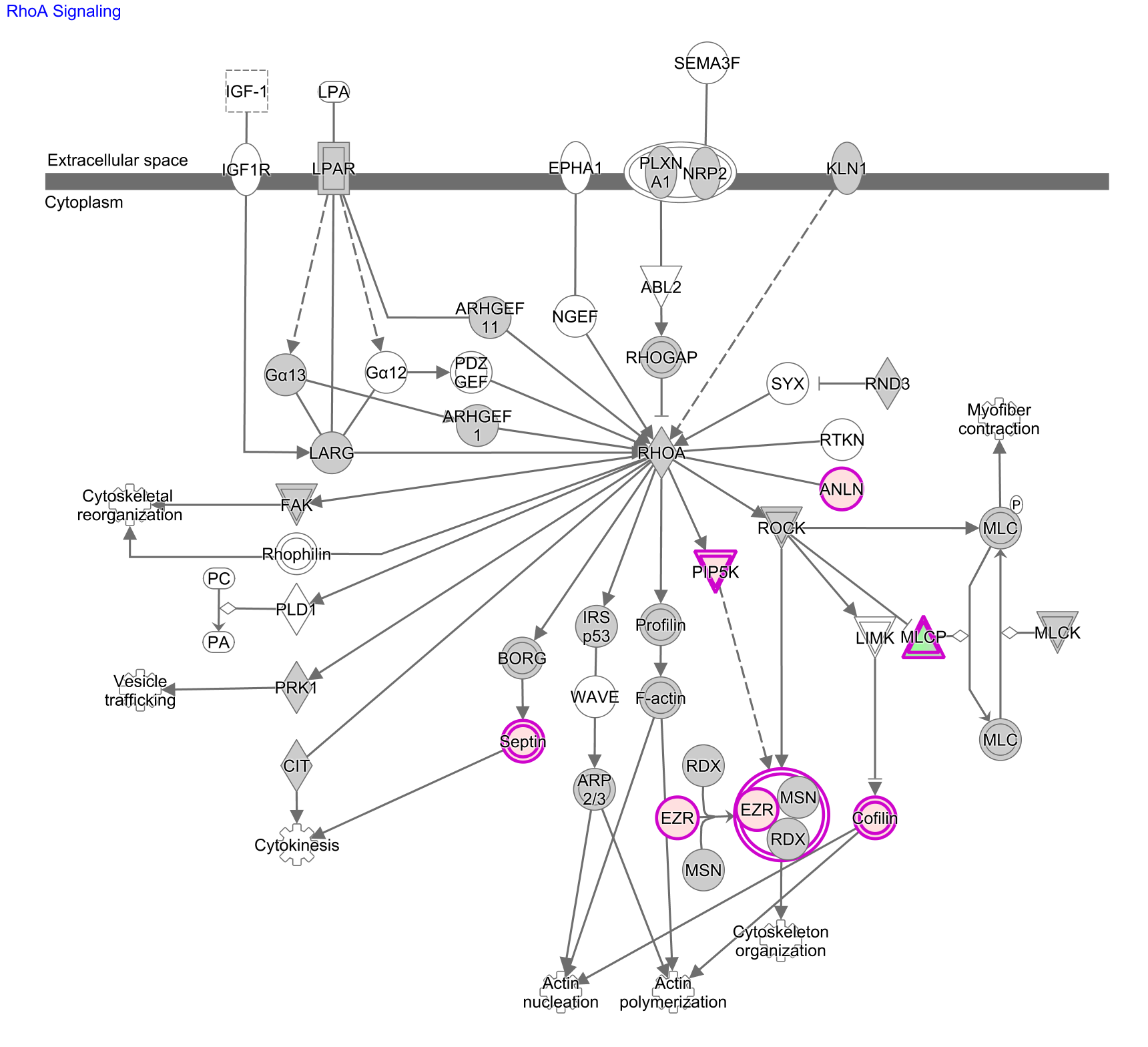

Supplement: Supplementary file 1 [file ijms-22-12176-s001.zip › Supplementary Figure S1.tif]

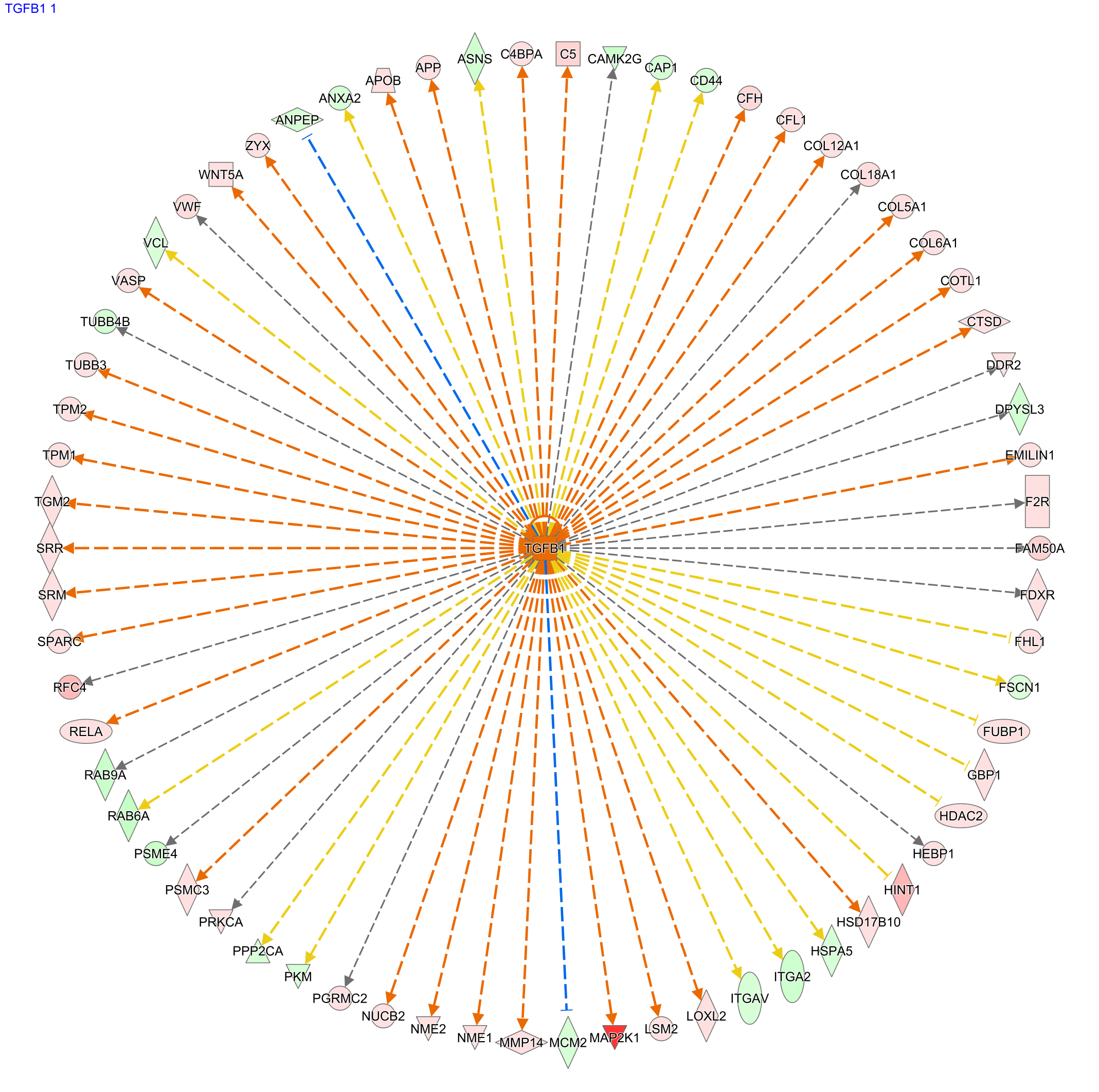

Supplement: Supplementary file 1 [file ijms-22-12176-s001.zip › Supplementary Figure S2.tif]

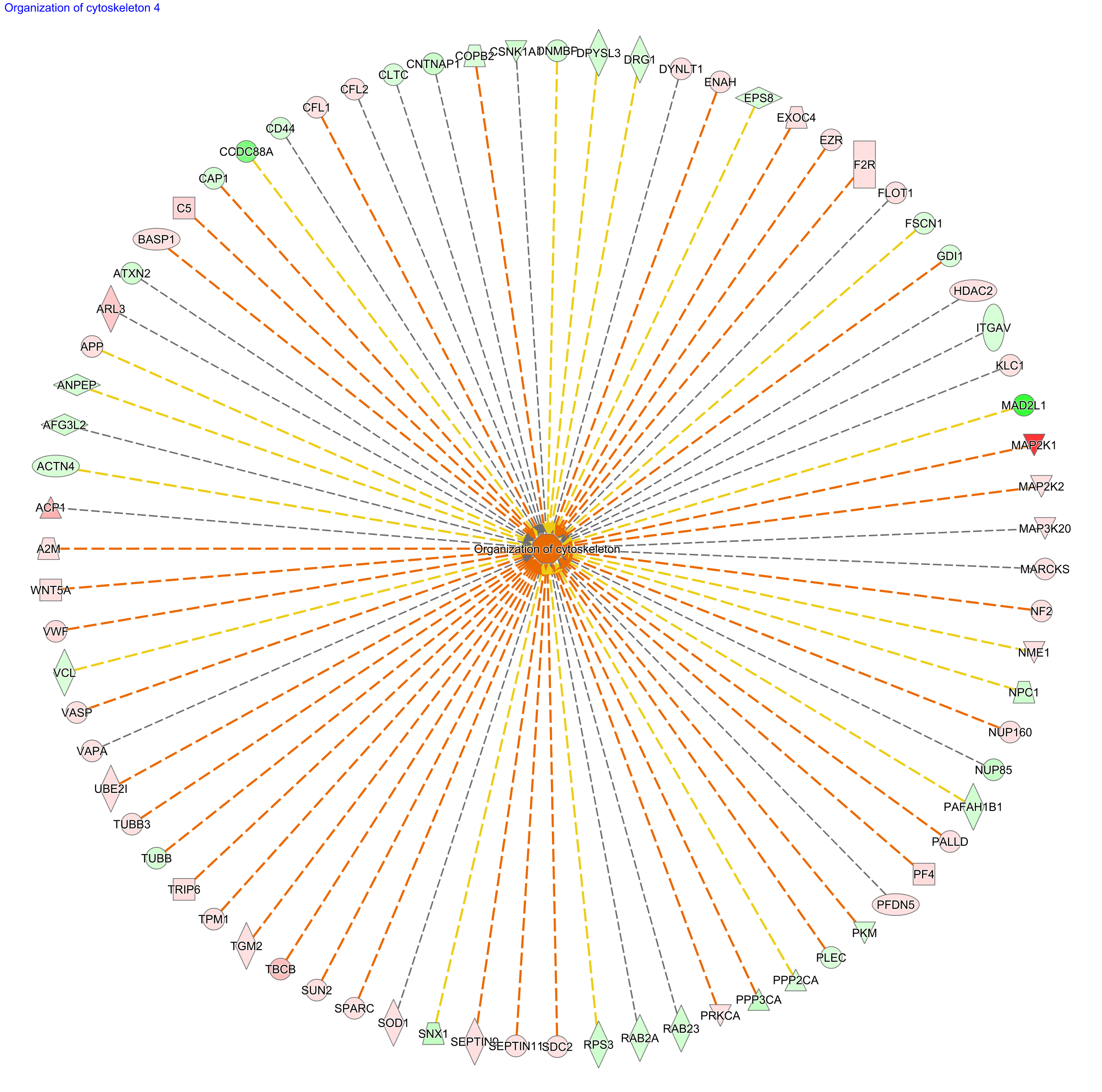

Supplement: Supplementary file 1 [file ijms-22-12176-s001.zip › Supplementary Figure S3.tif]

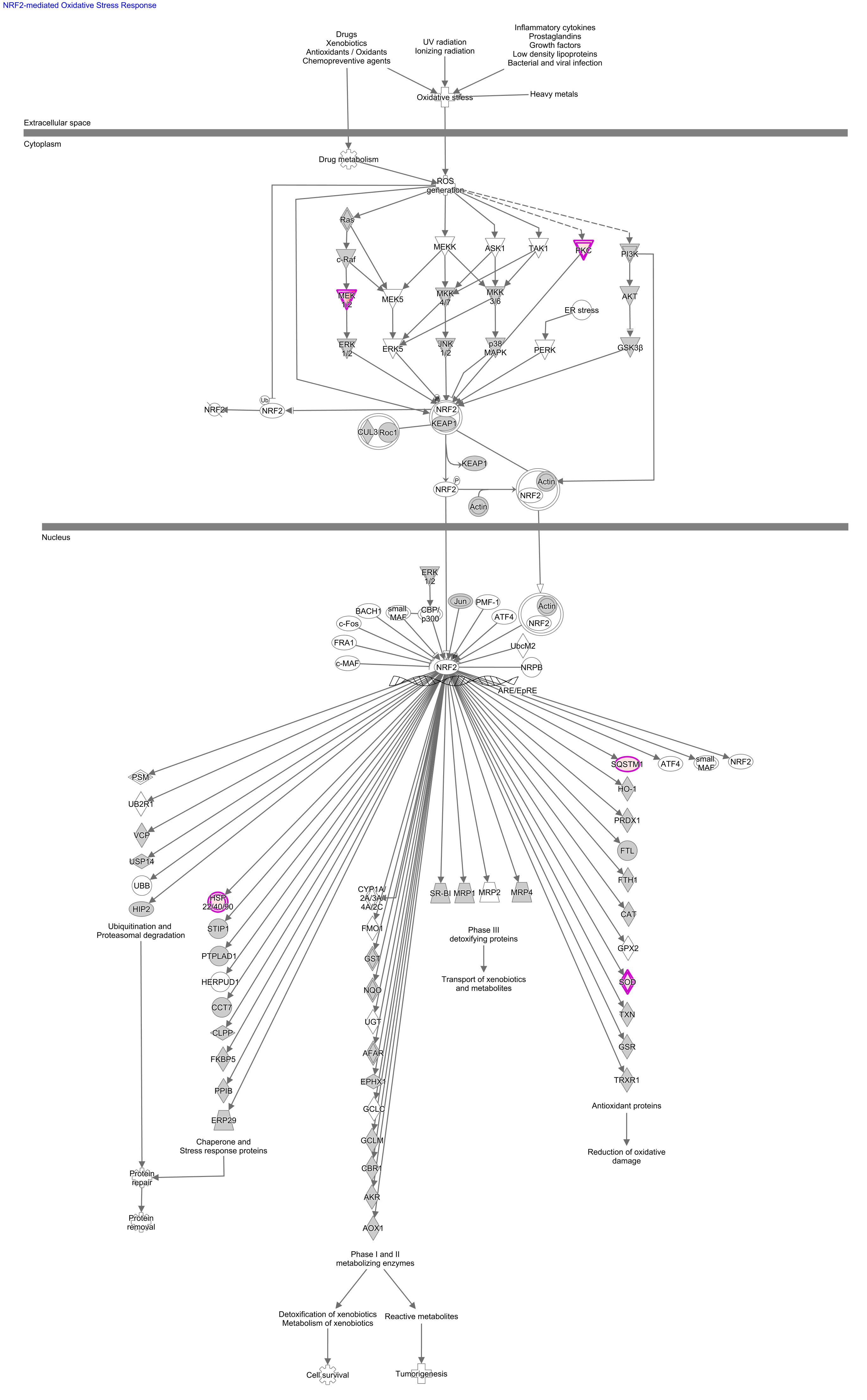

Supplement: Supplementary file 1 [file ijms-22-12176-s001.zip › Supplementary Figure S4.tif]
